# Impact of Food Additive Titanium Dioxide on Gut Microbiota Composition, Microbiota-Associated Functions, and Gut Barrier: A Systematic Review of In Vivo Animal Studies

**DOI:** 10.3390/ijerph18042008

**Published:** 2021-02-19

**Authors:** Emanuele Rinninella, Marco Cintoni, Pauline Raoul, Vincenzina Mora, Antonio Gasbarrini, Maria Cristina Mele

**Affiliations:** 1 UOC di Nutrizione Clinica, Dipartimento di Scienze Mediche e Chirurgiche, Fondazione Policlinico Universitario A. Gemelli IRCCS, Largo A. Gemelli 8, 00168 Rome, Italy; 2 Scuola di Specializzazione in Scienza dell’Alimentazione, Università di Roma Tor Vergata, Via Montpellier 1, 00133 Rome, Italy; marco.cintoni@gmail.com; 3 Dipartimento di Medicina e Chirurgia Traslazionale, Università Cattolica Del Sacro Cuore, Largo F. Vito 1, 00168 Rome, Italy; pauline.raoul1@gmail.com (P.R.); antonio.gasbarrini@unicatt.it (A.G.); mariacristina.mele@unicatt.it (M.C.M.); 4 UOC di Medicina Interna e Gastroenterologia, Dipartimento di Scienze Mediche e Chirurgiche, Fondazione Policlinico Universitario A. Gemelli IRCCS, Largo A. Gemelli 8, 00168 Rome, Italy; vincenzina.mora@policlinicogemelli.it; 5 UOSD di Nutrizione Avanzata in Oncologia, Dipartimento di Scienze Mediche e Chirurgiche, Fondazione Policlinico Universitario A. Gemelli IRCCS, Largo A. Gemelli 8, 00168 Rome, Italy

**Keywords:** dioxide titanium, TiO_2_, E171, CI 77891, food additive, gut microbiota, gut barrier, immunity, toxicity, diet

## Abstract

Background: Titanium dioxide (TiO_2_) is used as a food additive in pastries, sweets, and sauces. It is recognized as safe by food safety authorities, but in recent years, governments and scientists have raised concerns about its genotoxicity. This systematic review aims to assess the potential associations between food TiO_2_ exposure and microbiota composition and functions. Methods: A systematic literature search was performed up to December 2020 in PubMed, Web of Science, and Scopus databases. The PRISMA guidelines followed. The risk of bias was assessed from ARRIVE and SYRCLE tools. Results: A total of 18 animal studies were included (n = 10 mice, n = 5 rats, n = 2 fruit flies, n = 1 silkworm). Studies varied significantly in protocols and outcomes assessment. TiO_2_ exposure might cause variations in abundance in specific bacterial species and lead to gut dysfunctions such as a reduction in SCFAs levels, goblet cells and crypts, mucus production, and increased biomarkers of intestinal inflammation. Conclusions: Although the extrapolation of these results from animals to humans remains difficult, this review highlights the key role of gut microbiota in gut nanotoxicology and stimulates discussions on the safe TiO_2_ use in food and dietary supplements. This systematic review was registered at PROSPERO as CRD42020223968.

## 1. Introduction

Titanium dioxide (TiO_2_) is one of the main food additives used for its coloring and opacifying properties to improve the appearance and taste of processed foods. Food-grade TiO_2_ is found in over 900 food products such as pastries, sauces, ice-creams, candies, chocolates, and chewing gum. In foods, TiO_2_ is commonly reported as E171. It is also referred to as CI 77891 when used in cosmetics and toothpaste as a white colorant [1]. E171 consists of a wide range of particle TiO_2_ sizes and can contain up to 36% nanosized TiO_2_ particles, i.e., less than 100 nm in diameter [2,3]. Compared with their macroscopic counterparts, nanoparticles (NPs) can easier pass through the body’s cells and then into the bloodstream and internal organs such as liver, kidney, and lung tissues. Daily, the human dietary exposure dose of TiO_2_ NPs can reach one to four micrograms per kilogram body weight per day (µg per kg bw per day) [3]. In 1966, the Food and Drug Administration (FDA) approved the use of food-grade TiO_2_ referred to as INS171, specifying that the quantity of TiO_2_ must not exceed one percent by weight of the food [4]. In Europe, in 2006, the European Food Safety Authority (EFSA) authorized the use of E171 in food concluding that E171 is safe for consumers, having margins of safety (MoS) of 2.25 mg per kg bw per day [5,6]. However, TiO_2_ NPs raise health concerns among the scientific community and governments given their potential to cross the gut barrier and distribute to other organs eliciting immunological response. In June 2018, the EFSA evaluated four new in vivo and in vitro studies [7,8,9,10] assessing potential toxicities and reaffirmed the safety of E171 [11]. In April 2019, the French Agency for Food, Environmental and Occupational Health and Safety (ANSES) published a review suggesting a genotoxic and carcinogenic potential even if further in vivo mammalian studies are warranted to confirm or rule out these hypotheses [12]. As requested by the European Commission, EFSA provided urgent scientific and technical review regarding the opinion issued by ANSES [13]. The EFSA concluded that the latest ANSES opinion does not identify any major new findings that would overrule the conclusions made in the previous two scientific opinions in 2016 and 2018. The latest ANSES opinion reiterated the previously identified uncertainties and emphasized that there was still not enough data available to carry out a proper assessment of the risks associated with the food use of E171. EFSA considered this recommendation should be revisited once the ongoing work on the physicochemical characterization of E171 will be completed. In January 2020, France has adopted a decree to ban the use of E171 in foods as a precautionary measure to protect consumers’ health.

In a scientific context of “microbiota revolution”, potential health risks of TiO_2_ NPs and their impact on the intestinal tract and the gut microbiota are increasingly being studied. Gut microbiota is composed of millions of bacterial species that bi-directionally interact with the host in the intestinal tract, regulating the development of immune cells. Alterations in the abundance and composition of intestinal microbiota, known as dysbiosis, are associated with host health such as brain function, lipid metabolism, immune responses, and development of diseases [14]. Recent studies reported adverse effects of in vitro exposure of intestinal epithelial cells to E171 [9,15,16]. Indeed, TiO_2_ NPs could damage microvilli structure and alter epithelial integrity [17,18]. TiO_2_ NPs can be internalized and can cross the epithelial barrier to enter the bloodstream and potentially affect the function of distant organs, such as the liver [19]. Moreover, in vitro, NPs have the potential to negatively affect intestinal functions and gut homeostasis associated with gut microbiota [20]. New evidence from numerous recent animal studies has emerged highlighting the effects of various physiological doses of TiO_2_ NPs on gut microbiota composition and gut homeostasis. Such evidence has not yet been systematically reviewed. Hence, we sought to systematically review current evidence from in vivo animal models to disentangle the TiO_2_ effects on the gut microbiome composition and functions.

## 2. Methods

This systematic review is structured following the general principles published in the Preferred Reporting Items for Systematic Reviews and Meta-Analyses (PRISMA) guidelines [21]. The PRISMA checklist was detailed in Table S1. Full details of the search strategies were specified and documented in a protocol that was registered at PROSPERO (https://www.crd.york.ac.uk/PROSPERO; accessed on 24 December 2020) as CRD42020223968.

### 2.1. Eligibility Criteria

The eligibility criteria are outlined using the PICOS format (Table 1).

### 2.2. Data Sources and Search Strategy

The search was carried out on 1 December 2020 using three electronic databases, MEDLINE (via PubMed), ISI Web of Science, and Scopus. Multiple search terms are used including the microbiome, microflora, intestinal microbiota, gut microbiota, titanium dioxide, TiO_2_, and E171. The search string for each database is described in Table S2. Hand searching of eligible studies was done to find studies that may not have been found in the databases.

### 2.3. Study Selection

The study selection process was independently carried out by two reviewers (P.R.; E.R.). All articles generated from the electronic search were imported into Mendeley© (Elsevier, Amsterdam, The Netherlands), a references management software, and duplicates were removed. Titles and abstracts were screened for eligibility based on inclusion criteria. All titles assessed as ineligible were excluded. Differences in judgment during the selection process between the two reviewers were settled by discussion and consensus.

### 2.4. Data Extraction and Reporting

After full-text analysis, the following information was extracted from the included articles: title, author information, year of publication, type of study performed, assessed outcome/s, the animal model used, animal gender, age, and weight at baseline, administered dose, length of study, administration route, and main conclusions.

Data was reported using an Excel© (Microsoft Office, Redmond, WA, USA) spreadsheet specifically developed for this study. Each full-text article was retrieved, and any ineligible articles were excluded from the reasoning reported. Differences in judgment between two reviewers (P.R.; E.R.) were settled by discussion and consensus.

### 2.5. Quality Assessment

The quality of the included studies was assessed following the Animal Research Reporting of In Vivo Experiments (ARRIVE) guidelines [22]. These guidelines consist of the minimum information that animal research studies should include such as the number and specific characteristics of animals, details of housing and husbandry, experimental and statistical methods, reporting and interpretation of the results.

Moreover, SYRCLE’s risk of bias tool [23] was used to assess the risk of bias of animal studies. SYRCLE’s tool is an adapted version of the Risk of Bias tool provided by the Cochrane Collaboration. It consists of ten entries associated with selection bias, performance bias, detection bias, attrition bias, reporting bias, and other biases. Quality assessment was independently performed by two reviewers (P.R. and E.R.) and a consensus should be reached for discrepancies.

## 3. Results

### 3.1. Study Selection

**Figure 1:**
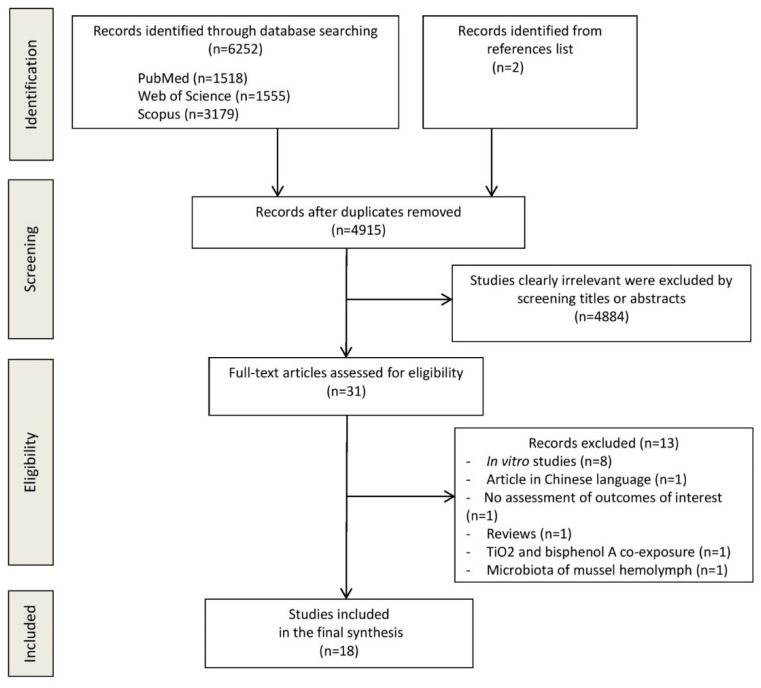
Preferred reporting items for systematic reviews and meta-analyses (PRISMA) flow diagram.

The flow diagram in Figure 1 displays the results of the literature search and study selection process. A total of 6254 studies were initially identified. After duplicate removal, 4915 studies remained for titles and abstracts screening. Thirteen studies were excluded for the following reasons: in vitro studies (n = 8) [9,19,24,25,26,27,28,29], no assessment outcomes of interest (n = 1) [30], microbiota of mussel hemolymph (n = 1) [31], review (n = 1) [32], Chinese language (n = 1) [33], TiO_2_ and bisphenol A co-exposure (n = 1) [34]. Eighteen studies [8,35,36,37,38,39,40,41,42,43,44,45,46,47,48,49,50,51] were identified for inclusion in the systematic review.

### 3.2. Study Characteristics

Included studies used different animal models: C57BL/6J mice (n = 5) [44,45,47,50,51], Sprague-Dawley rats (n = 3) [36,37,43], C57BL/6 (n = 3) [35,39,40], Wistar rats (n = 2) [8,48], Drosophila Melanogaster (n = 2) [42,46], CD-1 mice (n = 1) [38], ICR mice (n = 1) [49], and *Bombyx mori* (n = 1) [41]. Sample size ranged from 8 [43] to 80 animals [38]. Dose exposure ranged from 2 mg/day/kg body weight of TiO_2_ NPs [36,37,42,45] to 1 g/day/kg body weight of TiO_2_ NPs [39] and exposure period ranged from 24 h [39] to 100 days [8]. The characteristics of each included study are detailed in Table 2.

### 3.3. Quality Assessment

The detailed results of quality assessment are presented in Tables S3 and S4. First, the quality of the eighteen included animal studies was assessed through the ARRIVE guidelines. As a result, the included animal articles adequately provide an accurate title and abstract, a structured and thorough introduction, an ethical statement only for mammalian studies, and an adequate study design except for two studies [44,45] which are unclear. None of them justified the sample size, and consequently, the use of a too small number of animals may lead to a lack of experimental statistical significance given the use of too many animals may lead to unnecessary wastage of resources and ethical issues. Only one study did not clearly describe statistical methods [42]. Baseline characteristics (body weight, age, and gender) before treatment are reported in five of the total of studies [8,40,48,49,50]. For twelve studies [35,36,37,39,41,42,43,44,45,46,47,51], body weight was not specified, and for three studies [38,41,42], age was not reported. All studies adequately reported and interpreted their results in terms of numbers analyzed, outcomes, adverse events, interpretation, and generalizability.

Secondly, the risk of bias of the included animal studies was assessed using SYRCLE’s tool. In regards to sequence generation, in twelve out of eighteen studies, the allocation sequence was randomly generated and applied. However, in eleven out of 12 studies, the investigators did not describe the sequence generation process such as the use of a random number table or a computer random number generation. Only in the study of Zhang et al. [50], mice were randomly allocated into the control group and the TiO_2_ NPs’ group using a web-based randomization service. For all studies, it is not clear how animals were allocated to different groups. In addition, for all studies, all groups had similar characteristics at baseline. Regarding allocation concealment, the concealment was not clear for all studies. Indeed, no studies have explicated the concealed procedure when the investigators have allocated the animals to different groups. Moreover, all included studies have a high risk of performance bias. Indeed, the animals did not randomly house during the experiment and it is not clear whether the investigators did not blind from knowledge which intervention each animal received during the experiment. Additionally, overall, it is not specified whether the investigators did not select animals at random for outcome assessment. However, the outcome assessment methods are the same in both groups for all studies. In regards to attrition and reporting bias, the risk is low for all studies since the outcome data reported in each study was completed for each outcome. All primary outcomes have been reported. Finally, the studies did not report other problems that could result in a high risk of bias. As a conclusion, according to SYRCLE’s risk of bias tool, the quality of each study is debatable due to an inadequate or unclear randomization of allocation, housing and outcome assessment, and a lack of blinding. However, the studied population has similar characteristics at baseline making the sample homogenous and avoiding confounding bias. Moreover, in regards to the reporting of outcomes (complete outcome data reporting, adequate outcome reporting), the risk of bias is low.

### 3.4. Results

#### 3.4.1. TiO_2_ and gut Microbial Diversity

Alpha-diversity variations were measured in five studies [40,43,44,45,50]. Chao1—an estimate of species richness based on a vector or matrix of abundance data—did not significantly vary between exposed groups and controls groups in mice exposed to 100 mg/kg bw/day of TiO_2_ NPs for eight weeks [40], in pregnant rats exposed to 5 mg/kg bw/day of TiO_2_ NPs for 12 weeks [43], but decreased in mice exposed to 150 mg/kg bw/day of TiO_2_ NPs for 30 days (*p* = 0.0052) [50]. In regards to Shannon’s diversity—another index accounting for both abundance and evenness of the species with equal weighting given to abundant and rare species—no significant variations were observed between groups in mice exposed to 100 mg/kg bw/day of TiO_2_ NPs for eight weeks [40], in mice exposed to a diet containing 0.1% TiO_2_ NPs for three months [44], in mice exposed to 2, 10, 50 mg/kg bw/day of TiO_2_ NPs for three weeks [45], and in pregnant rats exposed to 5 mg/kg bw/day of TiO_2_ NPs for 13 days [43]. However, in the study of Zhang et al. [50], Shannon’s diversity decreased in mice exposed to 150 mg/kg bw/day of TiO_2_ NPs for 30 days (*p* = 0.0036) [50]. Finally, applying Simpson’s diversity index—another diversity index measuring richness and evenness in which more weighting is given to abundant species—in four out of the same studies [43,44,45,50], no significant variations were found except for the study of Zhang et al. [50] showing a significant increase after TiO_2_ NPs exposure (*p* = 0.0180).

#### 3.4.2. TiO_2_ and Abundance of Individual Microbial Species

In rodents, four studies showed an increase in Firmicutes abundance after TiO_2_ exposure compared with controls [35,39,49,51]. *Lactobacillus* was the most studied genus and significantly decreased in four studies [35,36,39,44] but increased in one study [45] after TiO_2_ NPs exposure compared with controls. Moreover, an increase in *Allobaculum* abundance was reported in one study [45] while a decrease was observed in another mice model [35]. Other variations in genera and family abundance after TiO_2_ exposure compared with controls are observed such as an increase in *Oscillospira* [35,51], *Turicibacter* [36], and Clostridiales [43], and a decrease in *Veillonella* [36], *Prevotella* [40,51], and *Dehalobacteriaceae* [43].

Bacteroidetes abundance could also be influenced by TiO_2_ exposure in rodent models. Three studies showed a decrease in Bacteroidetes levels [35,49,51] while one study reported an increase in Bacteroidetes levels [44]. Especially, TiO_2_ exposure could lead to an increase in *Bacteroides* [40], *Parabacteroides* [45], and a decrease in *Barnesiella* [49].

Actinobacteria phylum could decrease in abundance [35] after TiO_2_ exposure with a decrease in *Bifidobacterium spp* reported in two rodent studies [35,44]. Moreover, an increase in *Rhodococcus* abundance [40] and a decrease in *Adlercreutzia* levels [45] were observed.

In regards to other phyla, Proteobacteria could increase after TiO_2_ exposure, as reported in three studies [40,50,51], and Desulfovibrionaceae [51] and Verrucomicrobia could decrease, in particular in the *Akkermansia* genus [51].

All these findings observed in rodent models showed that TiO_2_ exposure could impact gut microbiota composition, although the variations in specific phyla and genera abundances remain to be elucidated with large sample size animal studies using the same dose and duration of TiO_2_ exposure.

In regards to animal models other than rodents, a model organism *Drosophila melanogaster* [42] showed that the exposure of 1, 2, and 200 mg/mL dietary of three different sizes of TiO_2_ NPs for five days did not inhibit the growth of gut bacteria in *Drosophila* larva or adults. On the other hand, a silkworm model [41] showed different gut microbiota compositional variations after intake of mulberry leaves soaked in 5 mg/L TiO_2_ NPs and naturally dried from the third day of fifth instar larvae until morning.

#### 3.4.3. TiO_2_ and SCFAs Levels

A total of six rodent studies reported between-group differences in fecal SCFA concentrations after different TiO_2_ NPs dose exposure and length of exposure. Three studies showed no significant variations in SCFAs levels [36,37,48] while two studies observed a decrease in SCFAs levels in mice treated with 0.1 weight percent of TiO_2_ NPs for eight weeks [35] and in mice treated with 50 mg TiO_2_/kg bw/day for three weeks [45]. Interestingly, one study [39] reported an increase of SCFAs in stools in mice exposed to 1 g/kg bw TiO_2_ for 14 days_._ This can be explained by an increase in SCFAs production or a decrease in absorption.

#### 3.4.4. TiO_2_ and Metabolism

A total of seven studies [36,37,39,42,43,49,50] showed significant metabolic variations in TiO_2_ exposed animals compared with controls. Lipopolysaccharides (LPS) proportionally increased in mice exposed to 2, 10, and 50 mg/kg bw/day of TiO_2_ for 30 days [36], in mice exposed to 10, 40, and 160 mg/kg bw/day of TiO_2_ for 28 days [49], and in mice exposed to 10 μL/g bw/day for eight weeks [51]. Interestingly, in TiO_2_-treated mice fed with a high-fat diet (HFD), LPS significantly increased compared with TiO_2_-treated mice fed with a high fiber diet (CHOW diet) [51]. Triglycerides levels (TG) levels increased in mice after exposure to 160 mg/kg bw/day of TiO_2_ for 28 days, while TG levels reduced in rats exposed to 10 and 50 mg/kg bw/day of TiO_2_ for 90 days. Moreover, glucose levels could increase after TiO_2_ exposure, as reported in two rodent model studies [43,49]. Interestingly, in Sprague-Dawley pregnant rats, exposure of 5 mg/kg/day of TiO_2_ NPs for 12 weeks could strengthen genes about type 2 diabetes mellitus related to function and lipid biosynthesis, compared with controls [43].

The two *Drosophila* model studies [42,46] reported contradictory results. One study showed no alterations of pupation cycle, weight, and lipid levels after 1, 2, and 200 mg/mL dietary TiO_2_ NPs of different sizes for five days while Richter and colleagues [46] demonstrated alterations of metabolic gut homeostasis with significant changes in pupation, time to pupation, reduction of body size, and glucose levels.

#### 3.4.5. TiO_2_ and Gut Barrier Permeability

Bettini et al. observed no significant changes in epithelial paracellular permeability in the E171 group in comparison to the controls [8]. Additionally, a previous study [48] found no effect compared with controls on mucin O-glycosylation in the small intestine of the rats following 7- or 60-day TiO_2_ exposure, regardless of TiO_2_ type (E171 and NM-105) or E171 dose tested (0.1 mg/kg bw/day and 10 mg/kg bw/day). Another study [39] showed that at 24 h post-gavage, MUC2 gene expression was lower in TiO_2_ NP-treated mice (1 g/kg bw/day) compared with controls but this trend was reversed from 48 h post-gavage to seven days with an elevated expression of mucin-2 for the rest of the study.

On the other hand, in mice exposed to 0.1 weight percent of TiO_2_, goblet cells and crypts significantly decreased compared to controls. Furthermore, three studies [45,49,51] reported a decrease in MUC2 gene expression in mice treated with TiO_2_ NPs. Yan et al. [49] also reported a reduction of mucus thickness in all exposed mice compared with controls. Interestingly, MUC2 gene expression and crypt length significantly decreased in TiO_2_-treated mice fed with HFD compared with TiO_2_-treated mice fed with CHOW diet [51].

#### 3.4.6. TiO_2_ and Inflammatory Responses

A total of ten studies have assessed levels of different gut microbiota associated biomarkers of mucosal immunity and intestinal inflammation such as interleukins (IL) levels, number of T reg cells, macrophages, and T helper cells. A reduction of T reg cells numbers was found in food-grade E171 treated mice after 100 days [8] and in mice exposed to a diet containing 0.1% TiO_2_ NPs for three months [44]. Inflammatory cytokines levels increased in exposed rodents compared with controls in the majority of studies: IL1 [49,51], IL2 [38], IL6 [8,36,45,49,51], IL10 [45], IL12 [35], IL17 [8,35], IL18 [8], as well as TNFα levels [45,49,51]. The production of macrophages and the expression of β defensin gene are also stimulated [45]. Interestingly, TiO_2_ NPs decreased the CD4+ T cells, T regs, and macrophages in the mesenteric lymph nodes and increased neutrophil gelatinase-associated lipocalin (LCN2) levels in mice aggravating the DSS-induced chronic colitis [44]. Moreover, IL-1 levels, IL-6 levels, TNFα levels increased in TiO_2_-treated mice fed with HFD compared with TiO_2_-treated mice fed CHOW diet [51]. All these results showed the potential involvement of TiO_2_ in the imbalances in intestinal and systemic immune responses.

## 4. Discussion

This systematic review of animal studies found that TiO_2_ dietary exposure might increase or decrease abundance in specific bacterial species, even if an overall impact on bacterial α-diversity has not been clearly demonstrated. Moreover, this review highlights that TiO_2_ exposure could lead to perturbations in intestinal metabolism, gut barrier integrity, and gut immunity.

The limited effect of TiO_2_ exposure on α-diversity of the gut microbiota was found in the majority of included studies. This could be explained by the short duration of the interventions, not exceeding three months. The lack of effects of different dietary interventions on gut microbiota diversity has been shown in previous systematic reviews investigating the effects of dietary patterns or dietary interventions—such as dietary fiber interventions or probiotics interventions—on gut microbiota [52]. Long-term studies are required to assess this hypothesis. In regards to bacterial abundances, in various included studies [35,39,44,49], significant compositional changes are reported after TiO_2_ exposure compared with controls. TiO_2_ exposure could lead to an alteration of the Firmicutes/Bacteroides ratio, a depletion of *Lactobacillus,* and enrichment of Proteobacteria [40,50]. Interestingly, these microbial variations are also found in studies investigating the effect of other food nanoparticles such as nano-Ag, ZnO, and SiO_2_ exposure [53]. *Lactobacillus* is a genus well-known to produce SCFAs, metabolites involved in host metabolism, while Proteobacteria might be overrepresented in inflammatory intestinal and extra-intestinal diseases. Indeed, this observed dysbiosis is also a hallmark of inflammatory bowel disease, colorectal cancer, or chronic metabolic disorders such as obesity [54].

The intestinal microbiota plays a key role in gastrointestinal functions such as the digestion and fermentation of indigestible polysaccharides, differentiation of the intestinal epithelium, and the maintenance of mucosal barrier integrity, including mucus characteristics. Mucus is a viscoelastic gel that separates the intestinal epithelium from the gut lumen. It consists of water and mucins, lipids, as well as epithelial and globets cells. Goblet cells are localized in the intestinal crypts and secrete proteins such as muc-2 (encoded by MUC2 gene). Intestinal bacteria influence the shaping of the mucus regulating LPS and SCFAs. Indeed, SCFAs—mainly butyrate—stimulate muc-2 protein production and influence mucus quality. Numerous studies [55,56,57] demonstrated that germ-free mice, comparing with conventionalized mice, were provided with an underdeveloped intestinal epithelium with decreased mucus production, intestinal epithelial cell differentiation, and villus thickness. These alterations could lead to an increased permeability allowing the passage of harmful intraluminal microorganisms and microbial toxins. These bidirectional interactions between gut microbiota composition and gut barrier functions could be impaired with TiO_2_ exposure. Indeed, in some included studies [35,45,49], TiO_2_ exposure could be associated with a reduction of SCFAs, a decrease of goblet cells and crypts, a reduction of mucus production with a lower MUC2 expression. These in vivo findings confirmed the results of in vitro studies demonstrating that TiO_2_ NPs could alter microvilli structure and epithelial integrity [19,24]. Particularly, in vivo and ex vivo, TiO_2_ NPs can cross the regular ileum and follicle-associated epithelium and alter the paracellular permeability of the ileum and colon epithelia, which is a sign of integrity alteration [58]. However, three studies [8,37,48] did not show significant changes in terms of epithelium permeability, SCFAs levels, and mucus barrier impairment. Considering the TiO_2_ dose exposure of the studies, we can hypothesize that these discrepancies could be due to dose exposure and healthy conditions of the animals at baseline.

TiO_2_ NPs also could interact with gut immunity. Indeed, a majority of included studies have assessed associations between TiO_2_ exposure and increased biomarkers of intestinal inflammation such as increased interleukins levels. Recent in vitro studies [19,27] found that TiO_2_ NPs could stimulate the production of pro-inflammatory cytokines. Moreover, in vivo, the number of T reg cells decreased after 100 days of TiO_2_ exposure [8]. T reg cells are well-known to limit gut inflammatory responses and prevent food allergy development [59]. Thus, long-term TiO_2_ exposure could have an immunosuppressive role by limiting the production of T reg cells. Interestingly, there are significant changes in terms of IL production, significantly aggravated in obese mice treated with TiO_2_ compared with non-obese mice [35,51]. This shows that TiO_2_ could exacerbate intestinal inflammation in mice affected by metabolic diseases such as obesity. Mu et al. [44] analyzed the effect of TiO_2_ NPs on DSS-induced chronic colitis in mice showing that DSS-induced chronic colitis worsened by chronic TiO_2_ NPs exposure with a reduction of immune cells such as CD4 + T cells and Tregs. Further studies are required to deepen the effects of TiO_2_ NPs on immunity responses, and specifically on the gut microbiota immune axis.

Overall, TiO_2_ exposure can raise concerns if we consider the cocktail effects of daily consumption of the different food additives. Indeed, other NPs present in food, emulsifiers, and artificial sweeteners have also dysbiotic effects on gut microbiota [60]. This cocktail effect raises particular concerns since the quantity of food additives is not detailed in the ingredient list, making impossible the calculation of the daily quantity of food additives. For example, chewing one piece of chewing gum can result in an intake of 1.5–5.1 mg of TiO_2_ NPs [61]. These concerns are even more important in children. Indeed, candies, gums, desserts, and beverages—products containing the highest levels of TiO_2_ NPs—are consumed two to four times higher for children than for adults [3]. A Dutch survey estimated a mean TiO_2_ NPs intake of 2.16 (2.13–2.26) mg/kg bw per day among children aged two to six years old, and a mean of 0.55 (0.52–0.58) among people aged 7–69 years old, with toothpaste, candy, coffee creamer, fine bakery wares, and sauces mostly contributing to the TiO_2_ daily intake [62]. Childhood is a key development time for the shape of the microbiota that can have considerable consequences in later life [63]. Although TiO_2_ consumption has considerably increased in the last few decades in Western countries and despite dietary composition having an impact on gut and overall health [64], the possible impacts of long term effects of TiO_2_ are still poorly understood.

This systematic review has some limitations. Although the majority of included studies have used rodent models, the methods of administration (gastric gavage, addition to drinking water, addition to food), TiO_2_ doses, and exposure durations differ between studies and do not allow pooling results. Thus, since some studies detect only a limited impact on the microbiota, others reporting various significant changes, it remains difficult to reach firm conclusions. Another limitation are the very high doses used in animal studies compared to the estimated daily intake in humans. Indeed, the amount of TiO_2_ consumed is estimated to 1 mg of TiO_2_/kg bw/day in adults in the United Kingdom and Germany, while the ingested quantity can exceed 3 mg of TiO_2_/kg bw/day in children [3,65]. Thus, the results from animal studies cannot be directly extrapolated to humans. Furthermore, only 15% of the 16S rRNA sequence dataset for the mouse microbiota are shared with humans [66]. Since randomized controlled studies are unethical, the use of germ-free mice inoculated with the human microbiota could be feasible to elucidate the impact of TiO_2_ NPs on gut bacteria that colonize the human intestine. Moreover, different dietary patterns such as HFD or high fiber diet should be evaluated to compare the impact on TiO_2_ NPs in healthy individuals with those in poor health.

## 5. Conclusions

In conclusion, in vivo consumption of TiO_2_ could alter the composition and the activity of intestinal bacteria, promoting an inflammatory environment in the gut and aggravating gut barrier impairment and immune responses in animals already affected by diseases such as colitis or obesity. Therefore, although these findings did not allow us to reach firm conclusions in humans, this systematic review highlights the key role of gut microbiota in nanotoxicology in the gut and stimulates discussions on the safe TiO_2_ use in food and dietary supplements.

## Figures and Tables

**Table 1:** PICOS criteria for inclusion of studies.

Criteria	Definition
Participants	Adult animals
Exposure	TiO_2_ NPs (rutile or anastase forms, with any size of nanoparticles)
Comparator	Any comparator
Outcomes	Primary outcomes - Between-group differences in α diversity of fecal microbiota at the end of the intervention: total number of observed operational taxonomic units (OTUs); Chao1 index; Shannon diversity index; Simpson diversity index; - Between-group differences in abundances of bacterial groups such as *Bifidobacterium* spp.; *Lactobacillus* spp.; *Akkermansia muciniphila*; *Faecalibacterium prausnitzii*; and *Ruminococcus bromii*. Secondary outcomes - Between-group differences in fecal SCFAs, Muc-2 gene expression, fasting blood glucose levels, lipid metabolism (such as LPS, HDL, LDL, and cholesterol levels); - Between-group differences in the inflammatory response (such as TNFα, IL-1α, IL-6, IL-10 levels, CD8+ T cells, CD4 + T cells, reg T cells production)
Study design	Peer-reviewed original animal experimental studies

The exclusion criteria were the following: (1) Non-English articles; (2) in vitro models; (3) review articles; (4) not fulfilling the inclusion criteria.

**Table 2:** Characteristics of included animal studies (listed by animal type).

Animal Species	First Author, Year	Sex	Age *	Weight *	Sample Size	TiO_2_ Particules Type and Size	Dose Exposure and Administration Route	Duration of Exposure	Significant Compositional Changes of Gut Microbiota (Compared with the Control Group)	Significant Effects on Microbiota-Associated Functions (Compared with the Control Group)
Mice Obese and non-obese C57BL/6	Cao, 2020 [35]	M	6 weeks	n.r.	N = 20 • low-fat diet (control) n = 5 • high fat diet n = 5 • high-fat diet + E171 n = 5 • high-fat diet + TiO_2_ NPs n = 5	E171, 112 nm TiO_2_ NPs, 33 nm	0.1 weight percent	8 weeks	In mice treated with TiO_2_ NPs + high-fat diet • ↑ Firmicutes • ↓ Actinobacteria and Bacteroidetes • ↓ *Bifidobacterium*, *Allobaculum* and *Lactobacillus* • ↑ *Oscillospira*	In mice treated with TiO_2_ NPs + high-fat diet: • ↓ SCFAs production • Loss of goblet cells and crypts • ↑ IL-12 • ↑ IL-17
Mice CD-1 (ICR)	Duan, 2010 [38]	F	n.r.	22 ± 2 g	N = 80 • control group (n = 20) • 62.5 mg/kg bw TiO_2_ (n = 20) • 125 mg/kg bw TiO_2_ (n = 20) • 250 mg/kg bw TiO_2_ (n = 20)	Anatase TiO_2_ NPs 5 nm	62.5, 125, 250 mg/ kg bw/ day TiO_2_ NPs via intragastric administration	30 days		• ↑ IL-2 activity in the exposed groups in a dose-dependent manner • ↓ CD3, CD4, and CD8 in the group treated with 250 mg/kg bwTiO_2_, • ↓ B cells and NK cells in all exposure groups
Mice C57/BL/6	Kurtz, 2020 [39]	F	7–8 weeks	n.r.	N = 48 • control group (n = 16) • TiO_2_ NPs (n = 32)	TiO_2_ NPs with irregular shapes	1 g/kg bw TiO_2_ NPs by oral gavage	24 h, 48 h, 7 days, and 14 days	• ↑ Firmicutes in the ileum at 14 days post-exposure • ↓ *Lactobacillus* spp. at 24 h	• ↑ SCFAs production in stools • Higher body weight • ↑ mucus production from 48 h post gavage to 7 days • ↑ IL-4 levels at 24 h
Mice C57BL/6	Li, 2018 [40]	M	8 weeks	22–26 g	N = 30 • control group (n = 10) • rutile TiO_2_ NPs (n = 10) • anatase TiO_2_ NPs (n = 10)	Anatase NPs in water 20.13 ± 0.18 nm Rutile NPs in water 15.91 ± 0.05 nm	100 mg/kg bw/day by oral gavage	28 days	• No decrease in overall microbiota diversity (Chao1 index, Shannon index, Simpson diversity index) • Shift of microbiota composition in a time-dependent manner • ↑ Proteobacteria by rutile TiO_2_ NPs but not by anatase TiO_2_ NPs • ↓ *Prevotella* by both TiO_2_ NPs • ↑ *Rhodococcus, Escherichia-Shigella* by rutile TiO_2_ NPs • ↑ *Bacteroides*, *Akkermansia* by anatase TiO_2_ NPs	In rutile TiO_2_ NPs—exposed mice: • Longer intestinal villi • Irregular arrangement of villus epithelial cells.
Mice C57BL/6J	Mu, 2019 [44]	F	3 weeks	n.r.	N = 20 • control group (n = 5) • NP10 (n = 5) • NP50 (n = 5) • NP100 (n = 5)	TiO_2_ NPs were added to the diet as an ingredient in the feed preparation process NP10: anatase; 10 nm NP50: anastase 50 nm NP100: anastase 100 nm	Diet containing 0.1% TiO_2_ NPs	3 months	• No significant bacterial diversity changes • ↑ Bacteroidetes in NP10 and NP50 treatment groups • ↓ Actinobacteria in NP10 and NP50 treatment groups • ↓ *Lactobacillus* and *Bifidobacterium* in NP10 and NP50 treatment groups	• Lower body weight in mice fed with NP10 and NP50 for 3 months • ↑ LCN2 levels in stools (a marker for intestinal inflammation) in NP10 and NP50-treated groups • Aggravation of DSS-induced chronic colitis • Aggravation of immune response • ↓ CD4 + T cells, regulatory T cells, and macrophages
Mice C57BL/6Jausb	Pinget, 2019 [45]	M	5–6 weeks	n.r.	N = 24 • control group (n = 6) • 2 mg TiO_2_/kg bw/day (n = 6) • 10 mg TiO_2_/kg bw/day (n = 6) • 50 mg TiO_2_/kg bw/day (n = 6)	Ti0_2_ NPs, 28 to 1158 nm	2, 10, 50 mg TiO_2_/kg bw/day orally administrated via drinking water	3 weeks	• Limited variations of bacterial diversity (Simpson, Shannon analyses), bacterial richness, and evenness in all exposed groups (although these trended toward decrease with increasing dose of TiO_2_) • ↑ *Parabacteroides* in TiO_2_-treated mice, at a dose of 50 mg TiO_2_/kg bw/day • ↑ *Lactobacillus* and *Allobaculum* in all exposed groups • ↓ *Adlercreutzia* and unclassified *Clostridiaceae* in mice treated with TiO_2_ at the doses of 10 and 50 mg TiO_2_/kg bw/day	In mice treated with 50 mg TiO_2_/kg bw/day: • ↓ SCFAs • ↓ TMA • ↓ crypt length In mice treated with 10 and 50 mg TiO_2_/kg bw/day: • ↓ MUC2 gene expression • ↑ expression of the β defensin gene • Infiltration of CD8+ T cells • ↑ production of macrophages (CD45 + F4/80 + CD8−Ly6 g−Iab +CD11b+ CD103−) • ↑inflammatory cytokines (IL-6, TNF-α and IL-10)
Mice Wild-type (C57BL/6J) and NLRP3-deficient	Ruiz, 2016 [47]	F	12–14 weeks	n.r.	N = 56 WT mice (n = 36) • water (n = 12) • 50 mg /day/kg body weight of TiO_2_ NPs (n = 12) • 500 mg /day/kg body weight of TiO_2_ NPs (n = 12) NLRP3-deficient mice (n = 20) • water (n = 10) • 500 mg /day/ kg body weight of TiO_2_ NPs (n = 10)	Suspension of TiO_2_ rutile NPs, 30–50 nm	50 and 500 mg/day/kg bw of rutileTiO_2_ NPs by oral gavage	8 days		• TiO_2_ NPs enhance intestinal inflammation in the DSS mouse model of colitis. • TiO_2_ proinflammatory effects required NLRP3 inflammasome activation when comparing WT with NLRP3-deficient mice
Mice ICR	Yan, 2020 [49]	M	Adult	18–20 g	N = 28 • control group (n = 7) • 10 mg/kg bw/day TiO_2_ NPs (n = 7) • 40 mg/kg bw/day TiO_2_ NPs (n = 7) • 160 mg/kg bw/day TiO_2_ NPs (n = 7)	Anatase TIO_2_ NPs, 20 nm	10, 40, 160 mg/ kg bw/day by oral gavage	28 days	• ↑ Firmicutes in all exposed groups • ↓ Verrucomicrobia in all exposed groups • ↓ Bacteroidetes at 160 mg/kg bw/day TiO_2_ • ↓ *Barnesiella* in all exposed groups in a dose-dependent manner • ↓ *Akkermansia* genus and *Porphyromonadaceae* family	• ↑ TG and glucose levels in the exposed group with 160 mg/kg bw/day TiO_2_ • ↑ LPS levels in all exposed groups. • ↑ IL-1α levels in the exposed group with 160 mg/kg bw/day TiO_2_ • ↑ IL-6 levels in all exposed groups • ↑ TNF-α levels in the exposed groups treated with 40 and 160 mg/kg/day TiO_2_ •↑ PKC protein at 40 mg/kg • Elevated TLR4 protein levels in the 40 and 160 mg/kg groups and P-P65 in all exposed groups • ↓ MUC2 expression at 160 mg/kg • ↓ intestinal mucus thickness in all exposed groups
Mice C57BL/6J	Zhang, 2020 [50]	M	7 weeks	20–24 g	N = 30 • control group (n = 15) • TiO_2_ NPs (n = 15)	TiO_2_ NPs, 21 nm	150 mg/kg bw/day by intragastric administration	30 days	• ↓ Richness and evenness of gut microbiota (decreased Shannon’s diversity, Chao, observed species and elevated Simpson’s diversity) • ↑ Proteobacteria	• No changes in body weight • Abnormal excitement on the enteric neurons • ↑ expression of TuJ1 (a neuronal marker of the peripheral nervous system) • No changes of IL-6 and IL-1β in the gut tissues
Mice C57BL/6J	Zhu, 2020 [51]	F	4–5 weeks	n.r.	N = 24 • control fed with CHOW diet (n = 6) • TiO_2_ NPs fed with CHOW diet (n = 6) • control fed with HFD (n = 6) • TiO_2_ NPs fed with HFD (n = 6)	TiO_2_ NPs, 30 ± 7 nm	10 μL/g bw/day via oral gavage	8 weeks	• ↑ Firmicutes to Bacteroidetes ratio in TiO_2_ NPs treated mice fed with HFD compared with both CHOW group and controls. • ↑ *Desulfovibrionaceae* in TiO_2_ NPs treated mice fed with CHOW or HFD compared with controls • ↑ *Ruminococcaceae* in TiO_2_ NPs treated mice fed with CHOW diet or HFD compared with controls • ↑ *Lachnospiraceae* in TiO_2_ NPs treated mice fed with CHOW compared with CHOW controls.	• ↓ crypt length in TiO_2_ groups compared with controls • ↓ muc2 expression in TiO_2_ groups compared with controls • ↓ MUC2 proteins levels in TiO_2_ groups compared with controls • ↑ LPS levels in TiO_2_ groups compared with controls with a significant increase in TIO_2_ mice fed HFD compared with TIO_2_ mice fed CHOW. • ↑ IL-1β, IL-6, and TNFα in TiO_2_ groups compared with controls with a significant increase in TIO_2_ mice fed HFD compared with TIO_2_ mice fed CHOW
Rats Wistar	Bettini, 2017 [8]	M	adult	175–200 g	First series of experiments N = 30 • control group (n = 10) • food-grade E171 (n = 10) • NM-105 (n = 10) Second series of experiments N = 34 • water (n = 11) treated with DMH • food-grade E171 (n = 11) treated with DMH • control (n = 12) water only	NM-105: Ti0_2_ NPs, 105 nm	10 mg/kg bw/day by intragastric gavage 10 mg/ kg bw/ day TiO_2_ NPs through drinking water	7 days 100 days		• No changes in epithelium permeability • Accumulation of dendritic cells in the immune cells of Peyer’s patches regardless of the TiO_2_ treatment • ↓ regulatory T cells • ↑ IL8, IL10, TNFα in food-grade E171 group after 100 days • At 7 days, no intestinal inflammation in E171 and NM-105 groups • Initiation of colon inflammation and pre-neoplastic lesions in the 100-day E171 group.
Rats Sprague-Dawley	Chen, 2019 [36]	M	3 weeks	n.r.	N = 12 • control group (n = 6) • TiO_2_ NPs (n = 6)	Anatase TiO_2_ NPs, 29 ± 9 nm	2, 10, 50 mg/ kg bw/ day TiO_2_ NPs via oral gavage	30 days	• ↑ *L. gasseri* in the high-dose group • ↑ *Turicibacter* in the low-dose group • ↑ *L. NK4A136_group* in the medium-dose group • ↓ *Veillonella* in all exposure groups	• Increase of N-acetylhistamine, caprolactam, and glycerophosphocholine • ↓ 4-methyl-5-thiazoleethanol, L-histidine, and L-ornithine • No significant changes in SCFAs levels • ↑ LPS production • ↑ IL-6 in the high-dose group • ↑ intestinal oxidative stress and inflammatory response
Rats Sprague-Dawley	Chen, 2020 [37]	M	3 weeks	n.r.	N = 12 • control group (n = 6) • TiO_2_ NPs (n = 6)	Anatase TiO_2_ NPs, 29 ± 9 nm	2, 10, 50 mg/ kg bw/ day TiO_2_ NPs via oral gavage	90 days		• No significant changes in SCFAs levels • ↓ TG levels in medium and high-dose groups • Significant serum lipophilic metabolites changes in the high-dose group with ↑ phosphatidylcholines and ↓ lysophosphatidylcholine and glycerophosphocholine levels • ↓ activity of the antioxidant enzyme SOD
Rats Pregnant Sprague-Dawley	Mao, 2019 [43]	F	12 weeks	n.r.	N = 8 • control group (n = 4) • TiO_2_ NPs (n = 4)	TiO_2_ NPs, 21 nm	5 mg/kg bw/day of TiO_2_ NPs	from the 5th to 18th day after pregnancy	• No significant changes of alpha-diversity (although the increasing trend in Shannon, and a significant change in Simpson index but no difference in Chao1) • ↓ *Ellin607* at GD 10 and GD 17 • Increase of *Clostridiales* at GD 10 • ↓ *Dehalobacteriaceae* at GD 17	• ↑ fasting blood glucose levels at GD 10 and GD 17 after exposure. • Strengthened genes about type 2 diabetes mellitus related function and lipid biosynthesis in the exposure group • Weakened taurine and hypotaurine metabolism in the exposure group
Rats Wistar	Talbot, 2018 [48]	M	Adult	175–200 g	First series of experiments N = 24 • control group (n = 8) • food-grade E171 (n = 8) • NM-105 (n = 8) Second series of experiments N = 30 • water (n = 10) • food-grade E171 (n = 10) • NM-105 (n = 10)	Food-grade E171, TiO_2_, 364 nm NM-105: TiO_2_ NPs, 105 nm	0.1 mg/kg bw/ day intragastric gavage 10 mg/kg bw/day through the drinking water	7 days 60 days		• No impact on the overall caecal composition of SCFAs (irrespectively of TiO_2_) • No effect on mucin O-glycosylation (irrespectively of TiO_2_) • Absence of mucus barrier impairment irrespectively of TiO_2_)
Fruit flies *Drosophila*	Liu, 2016 [42]	F	n.r.	n.r.	N = 45	10, 50, and 100 nm TiO_2_ NPs	1, 2 mg/mL and 200 mg/mL dietary TiO_2_ NPs of 3 different sizes	5 days	• No inhibition of the growth of symbiotic bacteria in the gut of *Drosophila* larva or adults	• No alteration of pupation cycle • No alteration of weight and lipid levels
Fruit flies *Drosophila Melanogaster*	Richter, 2018 [46]	n.r.	2 to 6 days	n.r.	N = 24 • control group (n = 6) • 5 ppM TiO_2_ NPs (n = 6) • 50 ppM TiO_2_ NPs (n = 6) • 500 ppM TiO_2_ NPs (n = 6)	TiO_2_ NPs, 30 nm	5 ppm, 50 ppm, 500 ppm of TiO_2_ NPs suspended in the food during cooking	From first instar larvae to adulthood		• Alteration of metabolic gut homeostasis with significant changes in pupation, time to pupation, time to emergence, body size, and glucose content.
Larvae of *Bombyx mori*	Li, 2020 [41]	n.r.	n.r.	n.r.	N = 16 • control group (n = 8) • TiO_2_ NPs (n = 8)	TiO_2_ NPs, 6–10 nm	Mulberry leaves soaked in 5 mg/L TiO_2_ NPs and naturally dried	From the 3rd day of fifth-instar larvae until morning	• ↑ *Staphylococcus*, *Lachnospiraceae*, *Pseudomonas*, *Sphingomonas*, *Kineococcus*, *Norank_f_bacteroidales,* • ↓ *Methylobacterium* and *Serratia*	

Abbreviations: ↓, decrease; ↑, increase; bw, body weight; DMH, dimethylhydrazine; F, female; GD, gestational day; HFD, high-fat diet; IFN, interferon; IL, interleukin; LPS, lipopolysaccharide; M, male; MUC2, oligomeric mucin-gel forming; NP, nano-particles; n.r., not reported; ppm, parts per million; SCFA, short chain fatty acid; SOD, superoxide dismutase; spp, species; TG, triglyceride; TLR4, toll-like receptor 4; TMA, trimethylamine. * Age at the start of the study.

## Data Availability

Not applicable.

## Supplementary Materials

The following are available online at https://www.mdpi.com/1660-4601/18/4/2008/s1, Table S1. PRISMA checklist; Table S2. Search strategy; Table S3. Reporting of in vivo experiments (ARRIVE) guideline assessment for the included animal studies; Table S4. SCYRCLE’s tool for assessing the risk of bias in animal studies.
