# Supplementary material for: Impact of Food Additive Titanium Dioxide on Gut Microbiota Composition, Microbiota-Associated Functions, and Gut Barrier: A Systematic Review of In Vivo Animal Studies
Source: Int J Environ Res Public Health. 2021 Feb 19;18(4):2008. doi: 10.3390/ijerph18042008 (PMC7922260; doi:10.3390/ijerph18042008)
Supplement: Supplementary file 1 [file ijerph-18-02008-s001.pdf]

SUPPLEMENTARY FILE

Table S1. PRISMA checklist.

TableS2. Search strategy.

Table S3. Reporting of in vivo experiments (ARRIVE) guideline assessment for the included animal studies.

Table S4. SCYRCLE's tool for assessing the risk of bias in animal studies.

Table S1. PRISMA checklist.

Section/topic	#	Checklist item	Reported on page #
**TITLE**			
Title	1	Identify the report as a systematic review, meta-analysis, or both.	**1**
**ABSTRACT**			
Structured summary	2	Provide a structured summary including, as applicable: background; objectives; data sources; study eligibility criteria, participants, and interventions; study appraisal and synthesis methods; results; limitations; conclusions and implications of key findings; systematic review registration number.	**1**
**INTRODUCTION**			
Rationale	3	Describe the rationale for the review in the context of what is already known.	**1-2**
Objectives	4	Provide an explicit statement of questions being addressed with reference to participants, interventions, comparisons, outcomes, and study design (PICOS).	**2**
**METHODS**			
Protocol and registration	5	Indicate if a review protocol exists, if and where it can be accessed (e.g., Web address), and, if available, provide registration information including registration number.	**2**
Eligibility criteria	6	Specify study characteristics (e.g., PICOS, length of follow-up) and report characteristics (e.g., years considered, language, publication status) used as criteria for eligibility, giving rationale.	**2-3**
Information sources	7	Describe all information sources (e.g., databases with dates of coverage, contact with study authors to identify additional studies) in the search and date last searched.	**3**
Search	8	Present full electronic search strategy for at least one database, including any limits used, such that it could be repeated.	**Table S2**
Study selection	9	State the process for selecting studies (i.e., screening, eligibility, included in systematic review, and, if applicable, included in the meta-analysis).	**3**
Data collection process	10	Describe method of data extraction from reports (e.g., piloted forms, independently, in duplicate) and any processes for obtaining and confirming data from investigators.	**3-4**
Data items	11	List and define all variables for which data were sought (e.g., PICOS, funding sources) and any assumptions and	**Table 1**

		simplifications made.	
Risk of bias in individual studies	12	Describe methods used for assessing risk of bias of individual studies (including specification of whether this was done at the study or outcome level), and how this information is to be used in any data synthesis.	**4**
Summary measures	13	State the principal summary measures (e.g., risk ratio, difference in means).	**-**
Synthesis of results	14	Describe the methods of handling data and combining results of studies, if done, including measures of consistency (e.g., I^2) for each meta-analysis.	**-**
**Section/topic**	**#**	**Checklist item**	**Reported on page #**
Risk of bias across studies	15	Specify any assessment of risk of bias that may affect the cumulative evidence (e.g., publication bias, selective reporting within studies).	**-**
Additional analyses	16	Describe methods of additional analyses (e.g., sensitivity or subgroup analyses, meta-regression), if done, indicating which were pre-specified.	**-**
**RESULTS**			
Study selection	17	Give numbers of studies screened, assessed for eligibility, and included in the review, with reasons for exclusions at each stage, ideally with a flow diagram.	**4 Figure 1**
Study characteristics	18	For each study, present characteristics for which data were extracted (e.g., study size, PICOS, follow-up period) and provide the citations.	**5-11 Table 2**
Risk of bias within studies	19	Present data on risk of bias of each study and, if available, any outcome level assessment (see item 12).	**Tables S3 S4**
Results of individual studies	20	For all outcomes considered (benefits or harms), present, for each study: (a) simple summary data for each intervention group (b) effect estimates and confidence intervals, ideally with a forest plot.	**-**
Synthesis of results	21	Present results of each meta-analysis done, including confidence intervals and measures of consistency.	**-**
Risk of bias across studies	22	Present results of any assessment of risk of bias across studies (see Item 15).	**-**
Additional analysis	23	Give results of additional analyses, if done (e.g., sensitivity or subgroup analyses, meta-regression [see Item 16]).	**-**
**DISCUSSION**			
Summary of evidence	24	Summarize the main findings including the strength of evidence for each main outcome; consider their relevance to key	**12-14**

		groups (e.g., healthcare providers, users, and policy makers).	
Limitations	25	Discuss limitations at study and outcome level (e.g., risk of bias), and at review-level (e.g., incomplete retrieval of identified research, reporting bias).	**16**
Conclusions	26	Provide a general interpretation of the results in the context of other evidence, and implications for future research.	**15-17**
**FUNDING**			
Funding	27	Describe sources of funding for the systematic review and other support (e.g., supply of data); role of funders for the systematic review.	**17**

Table S2. Search strategy performed on 1st December 2020

**MEDLINE**	**Search terms**	**Results**
#1	(titanium AND dioxide) OR titanium OR TIO2 OR E171 OR E-171	79,549
#2	microbiota[MeSH Terms] OR microbiome OR (intestinal AND flora) OR (gut AND flora) OR microbial	494,198
#3	#1 AND #2	1,518
**WEB OF SCIENCE**	**Search terms**	**Results**
#1	(titanium AND dioxide) OR titanium OR TIO2 OR E171 OR E-171	247,984
#2	microbiota OR microbiome OR (intestinal AND flora) OR (gut AND flora) OR microbial	403,547
#3	#1 AND #2	1,555
**SCOPUS**	**Search terms**	**Results**
#1	**TITLE-ABS-KEY:** (titanium AND dioxide) OR titanium OR TIO2 OR E171 OR E-171	484,117
#2	**TITLE-ABS-KEY:** microbiota OR microbiome OR (intestinal AND flora) OR (gut AND flora) OR microbial	664,024
#3	#1 AND #2	3,179

Table S3. Reporting of in vivo experiments (ARRIVE) guideline assessment for the included animal studies.

First Author, Year of Publication	1. Title	2. Abstract	3. Background	4. Objectives	5. Ethical statement	6. Study design	7. Experimental procedures	8. Experimental animals	9. Housing and husbandry	10. Sample size	11. Animal allocation	12. Outcome determination	13. Statistical methods	14. Baseline data	15. Numbers analyzed	16. Outcomes	17. adverse events	18. Interpretation	19. Generalisability	20. Funding	STATUS
Bettini, 2017	Yes	Yes	Yes	Yes	Yes	Yes	Yes	Yes	Yes	No sample size calculation	unclear	Yes	Yes	Yes	Yes	Yes	not reported	Yes	Yes	Yes	included
Cao, 2020	Yes	Yes	Yes	Yes	Yes	Yes	Yes	Yes	Yes	No sample size calculation	Yes	Yes	Yes	Weight not indicated	Yes	Yes	not reported	Yes	Yes	Yes	included
Chen, 2019	Yes	Yes	Yes	Yes	Yes	Yes	Yes	Yes	Yes	No sample size calculation	Yes	Yes	Yes	Weight not indicated	Yes	Yes	not reported	Yes	Yes	Yes	included
Chen, 2020	Yes	Yes	Yes	Yes	Yes	Yes	Yes	Yes	Yes	No sample size calculation	Yes	Yes	Yes	Weight not indicated	Yes	Yes	not reported	Yes	Yes	Yes	included
Duan, 2010	Yes	Yes	Yes	Yes	Yes	Yes	Yes	Yes	Yes	No sample size calculation	Yes	Yes	Yes	Age not indicated	Yes	Yes	not reported	Yes	Yes	Yes	included
Kurtz, 2020	Yes	Yes	Yes	Yes	Yes	Yes	Yes	Yes	Yes	No sample size calculati	Yes	Yes	Yes	Weight not indicated	Yes	Yes	not reported	Yes	Yes	Yes	included

										on											
Li, 2018	Yes	Yes	Yes	Yes	Yes	Yes	Yes	Yes	Yes	No sample size calculation	Yes	Yes	Yes	No	Yes	Yes	not reported	Yes	Yes	Yes	included
Li, 2020	Yes	Yes	Yes	Yes	No specific permissions were required for the sampling of silkworms	Yes	Yes	Yes	Yes	No sample size calculation	Yes	Yes	Yes	No	Yes	Yes	Yes	Yes	Yes	Yes	included
Liu, 2016	Yes	Yes	Yes	Yes	No specific permissions were required for the sampling of fruit flies	Yes	Yes	Yes	Yes	unclear total number of animals used in each experiment and the number of animals in each experimental group	unclear	Yes	unclear	No	Yes	Yes	not reported	Yes	unclear	Yes	included
Mao, 2019	Yes	Yes	Yes	Yes	Yes	Yes	Yes	Yes	Yes	No sample	Yes	Yes	Yes	Weight not	Yes	Yes	not reported	Yes	Yes	Yes	included

										size calculation				indicated							
Mu, 2019	Yes	Yes	Yes	Yes	Yes	unclear	Yes	Yes	unclear	unclear	unclear	Yes	Yes	Yes	Yes	Yes	Yes	Yes	Yes	Yes	included
Pinget, 2019	Yes	Yes	Yes	Yes	Yes	unclear	Yes	Yes	Yes	unclear	unclear	Yes	Yes	Unclear	unclear	Yes	not reported	Yes	Yes	Yes	included
Richter, 2018	Yes	Yes	Yes	Yes	No specific permissions were required for the sampling of fruit flies	Yes	Yes	Yes	Yes	No sample size calculation	Yes	Yes	Yes	Unclear	Yes	Yes	not reported	Yes	Yes	Yes	included
Ruiz, 2016	Yes	Yes	Yes	Yes	Yes	Yes	Yes	Yes	Yes	No sample size calculation	Yes	Yes	Yes	Weight not indicated	Yes	Yes	not reported	Yes	Yes	Yes	included
Talbot, 2018	Yes	Yes	Yes	Yes	Yes	Yes	Yes	Yes	Yes	No sample size calculation	Yes	Yes	Yes	Yes	Yes	Yes	not reported	Yes	Yes	Yes	included
Yan, 2020	Yes	Yes	Yes	Yes	Yes	Yes	Yes	Yes	Yes	No sample size calculation	Yes	Yes	Yes	Yes	Yes	Yes	not reported	Yes	Yes	Yes	included
Zhang, 2020	Yes	Yes	Yes	Yes	Yes	Yes	Yes	Yes	Yes	No sample size	Yes	Yes	Yes	Yes	Yes	Yes	not reported	Yes	Yes	Yes	included

										calculation											
Zhu, 2020	Yes	Yes	Yes	Yes	Yes	Yes	Yes	Yes	Yes	No sample size calculation	Yes	Yes	Yes	Yes	Yes	Yes	not reported	Yes	Yes	Yes	included

Table S4. SCYRCLE's tool for assessing the risk of bias in animal studies.

(+) indicates low risk of bias (-) indicates high risk of bias (?) indicates unclear risk of bias

First author, Year of publication	1. Sequence generation	2. Baseline characteristics	3. Allocation concealment	4. Random housing	5. Blinding (performance bias)	6. Random outcome assessment	7. Adequate blinding (detection bias)	8. Complete outcome data reporting	9. Adequate outcome reporting	10. Absence of other sources of bias
Bettini, 2017	?	+	?	-	?	?	+	+	+	+
Cao, 2020	?	+	?	-	?	?	+	+	+	+
Chen, 2019	?	+	?	-	?	?	+	+	+	+
Chen, 2020	?	+	?	-	?	?	+	+	+	+
Duan, 2010	?	+	?	-	?	?	+	+	+	+
Kurtz, 2020	-	+	?	-	?	?	+	+	+	+
Li, 2018	?	+	?	-	?	?	+	+	+	+
Li, 2020	-	+	?	-	?	?	+	+	+	+
Liu, 2016	-	+	?	-	?	?	+	+	+	+
Mao, 2019	?	+	?	-	?	?	+	+	+	+
Mu, 2019	-	+	?	-	?	?	+	+	+	+
Pinget, 2019	?	+	?	-	?	?	+	+	+	+
Richter, 2018	-	+	?	-	?	?	+	+	+	+

Ruiz, 2016	?	+	?	-	?	?	+	+	+	+
Talbot, 2018	?	+	?	-	?	?	+	+	+	+
Yan, 2020	?	+	?	-	?	?	+	+	+	+
Zhang, 2020	+	+	?	-	?	?	+	+	+	+
Zhu, 2020	?	+	?	-	?	?	+	+	+	+
